# Introducing Two New Bacteriophages Isolated Using *Arthrobacter globiformis* : BlueShadow and Schaffner

**DOI:** 10.17912/micropub.biology.001938

**Published:** 2026-01-06

**Authors:** Madeline Dojs, Christine Fleischacker, Celia Brekken, Ethan Emineth

**Affiliations:** 1 Biology, University of Mary, Bismarck, North Dakota, United States

## Abstract

Two newly discovered phages, BlueShadow and Schaffner, were isolated from soil in Bismarck, ND using the host *Arthrobacter globiformis B-2979* . Based on gene content similarity, BlueShadow is assigned to actinobacteriophage cluster AY, while Schaffner is assigned to cluster AZ1. Both phages encode a putative integrase that is conserved within their respective clusters, implying a temperate lifestyle.

**Figure 1. BlueShadow is on the left, Schaffner is on the right:**
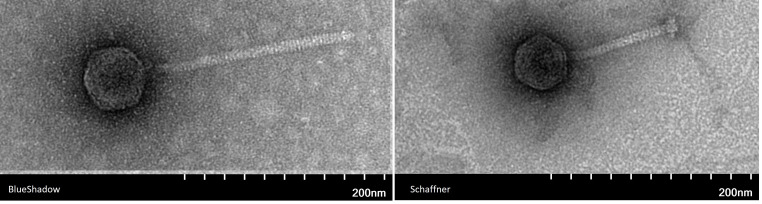
BlueShadow is on the left, Schaffner is on the right. BlueShadow mean capsid length = 61.28 nm (n=3), mean tail length = 209.04nm (n=3). Schaffner mean capsid length = 52.34nm (n=3), mean tail length = 126.52nm (n=3).

## Description

Bacteriophages are viruses that exclusively infect bacteria. Since their discovery, several phages have been used to combat antibiotic-resistant bacterial infections (Hatful, 2022). Here, we detail the isolation and characteristics of two newly identified bacteriophages, BlueShadow and Schaffner, found to infect *Arthrobacter globiformis* B-2979, a widespread soil bacterium not known to be associated with human disease.

Phages BlueShadow and Schaffner were isolated from soil samples collected in Bismarck, ND.&nbsp; The soil sample for BlueShadow was dark and moist and collected next to a stone retaining wall (GPS: 46.72623 N, 100.75309 W), whereas Schaffner was extracted from a soil sample where perennials were unsuccessfully cultivated (GPS: 46.88773 N, 100.70426 W). Both soil samples were washed with peptone-yeast extract-calcium (PYCa) liquid medium, filtered through a 0.22-µm filter, and mixed with a soft agar containing *A. globiformis* B-2979, overlaid onto PYCa agar, and incubated at 30°C for 48 hours (Zorawik et al).&nbsp;After 48 hours, BlueShadow displayed an average plaque size of 3.06 mm +/- .05mm (n=3), and Schaffner displayed an average plaque size of 0.9 mm +/- .1mm (n=3). Both phages were purified through three rounds of plating. Transmission electron microscopy with negative staining (1% uranyl acetate) revealed that both phages exhibited siphovirus morphology (Figure 1).

DNA was isolated from BlueShadow and Schaffner using the Promega Wizard DNA cleanup kit. The genome was sequenced using an Illumina NextSeq 1000 (XLEAP-P1 kit), with libraries prepped using the NEB Ultra II FS kit yielding 4,697,068 single-end 100-bp reads for BlueShadow and 4,803,542 single-end 100-bp reads for Schaffner. Raw reads were trimmed with cutadapt 4.7 (using the option: –nextseq-trim 30) and filtered with skewer 0.2.2 (using the options: -q 20 -Q 30 -n -l 50) prior to assembly.(Martin, Jiang et al, Wick et al, Gordon et al) The resulting genome of BlueShadow was 52,454 bp long with a 3′ sticky overhang of 9bp and a G+C content of 62.7%: Whereas the genome of Schaffner is 43,610 bp long, with 3' sticky overhang of 11bp and a GC content of 68.1% as previously described (Russell DA). BlueShadow was assigned to cluster AY and Schaffner was assigned to cluster AZ1 based on gene content similarity of at least 35% to phages in the Actinobacteriophage Database, phagesdb (https://phageDB.org) (Pope et al., 2017; Russell and Hatfull, 2017). 

Genome annotation was performed using DNA Master (cobamide2.bio.pitt.edu) and PECAAN (discover.kbrinsgd.org). Both programs use Glimmer-v3.02 (Delcher, 2007), Genemark-v3.25 (Besemer and Borodovsky, 2005), Phamerator (Cresawn, 2011), and Starterator (http://phages.wustl.edu/starterator/ref) for the prediction of potential open-reading frames (ORFs). ARAGORN v1.2.41 (Laslett, 2004) and tRNA scan-SE 2.0 (Lowe, 1997), were used to detect the presence of tRNAs. Gene functions were predicted using BLASTp (Altschul et al, 1990) searches against the Actinobacteriophage and NCBI non-redundant databases as well as HHPred v3.18 (Söding, 2005) searches against the PDB mmCIF70, Pfam-A, and NCBI Conserved Domain databases. All software was used with default parameters. 

The genome of BlueShadow is composed of 93 proposed protein-coding genes, 49 of which have a known protein function. The genome of Schaffner contains 66 proposed protein-coding genes, 36 of which have a known protein function. Both phages encode for integrase functions suggesting a temperate life cycle; BlueShadow encodes two tyrosine integrases (37 & 40), whereas Schaffner encodes a serine integrase (50). BlueShadow encodes a large terminase across two ORFs (ATPase domain, BlueShadow 2; Nuclease domain, BlueShadow 3), as is characteristic of most AY phages (Gavin et al, 2024).&nbsp; Endolysins are well-conserved across cluster AZ1, but the location is not conserved. In Schaffner, this endolysin is found in the second half of the genome (Schaffner 57) similar to phage Yang (Dojs et al, 2024). 

Nucleotide sequence accession numbers 

The GenBank and Sequence Read Archive (SRA) accession numbers for BlueShadow are PV876918 and SRX27501634 , respectively. The GenBank and SRA accession numbers for Schaffner are PV876958 and SRX27501638 , respectively.
